# Silicon nanowire and carbon nanotube hybrid for room temperature multiwavelength light source

**DOI:** 10.1038/srep16753

**Published:** 2015-11-23

**Authors:** Maria Josè Lo Faro, Cristiano D’Andrea, Elena Messina, Barbara Fazio, Paolo Musumeci, Riccardo Reitano, Giorgia Franzò, Pietro Giuseppe Gucciardi, Cirino Vasi, Francesco Priolo, Fabio Iacona, Alessia Irrera

**Affiliations:** 1 IPCF-CNR, viale F. Stagno d’Alcontres 37, 98158 Messina, Italy; 2 MATIS IMM-CNR, via S. Sofia, 64, 95123 Catania, Italy; 3 Dipartimento di Fisica e Astronomia, Università di Catania, via S. Sofia, 64, 95123 Catania, Italy; 4 Scuola Superiore di Catania, Università di Catania, via Valdisavoia, 9, 95123 Catania, Italy

## Abstract

The realization of an innovative hybrid light source operating at room temperature, obtained by embedding a carbon nanotube (CNT) dispersion inside a Si nanowire (NW) array is reported. The NW/CNT system exhibits a peculiar photoluminescence spectrum, consisting of a wide peak, mainly observed in the visible range, due to quantum confined Si NWs, and of several narrower IR peaks, due to the different CNT chiralities present in the dispersion. The detailed study of the optical properties of the hybrid system evidences that the ratio between the intensity of the visible and the IR emissions can be varied within a wide range by changing the excitation wavelength or the CNT concentration; the conditions leading to the prevalence of one signal with respect to the other are identified. The multiplicity of emission spectra obtainable from this composite material opens new perspectives for Si nanostructures as active medium in light sources for Si photonics applications.

In the last years the scientific community has devoted an increasing interest towards nanostructured materials. In this field, Si nanowires (NWs) represent one of the most promising systems; indeed, they are able to confine excitons in two directions, so that both their electrical and optical properties are dramatically modified with respect to bulk Si, which makes them suitable candidates to become the building blocks for innovative electronic^1^, photovoltaic^2^ and sensing devices^3^. On the other hand, light emission from Si NWs is still a scarcely reported phenomenon; the main reason for this lack of results is that the most popular techniques used for NW synthesis, based on the vapor-liquid-solid mechanism or on top-down lithographic processes^4^,^5^, can hardly produce wires having a suitable size to exhibit strong quantum confinement effects. Recently, it has been demonstrated that a strong and tunable light emission under both optical and electrical excitation, related to the occurrence of quantum confinement phenomena, can be observed at room temperature from ultrathin Si NWs obtained by a properly modified process of metal assisted chemical etching of Si wafers^6^,^7^, this achievement opened new and unexpected perspectives for this material, suggesting that it may play an important role also as active medium in light sources to be employed in Si photonics^6^,^7^,^8^,^9^,^10^.

On the other hand, also carbon nanotubes (CNTs) have become a key material of nanotechnology, due to the intriguing properties related with their quasi-1D nature^11^,^12^. Single CNTs are able to outperform Cu in terms of electrical^13^ and thermal conductivity^14^, and exhibit an extraordinary mechanical strength^15^ and rich optical spectra^16^,^17^,^18^. Individual or small bundles of semiconducting single wall CNTs (SWCNTs) are characterized by a photoluminescence (PL) signal in the IR range; their emission wavelength varies with the chirality, making them an interesting tunable photon source. Recently, a high-efficiency SWCNT LED, consisting of a single tube with one end connected to a source and the other to a drain, has been proposed^19^. Also CNT thin films exhibit interesting properties; for instance, electroluminescence from CNT macroassemblies has been reported^20^, suggesting that also simpler photonic applications than those based on single tubes are possible for this material.

The idea of putting together NWs and CNTs has been already demonstrated and it appears to be a very promising strategy for a variety of applications. Hybrid solar cells, where a thin film of double-walled CNTs forms a heterojunction with a Si NW array, have been recently reported^21^. These cells show high energy conversion efficiencies of about 6%^22^. CNT-metal nanoparticle hybrids have been used to enhance nucleation and growth of Si NWs and thus to synthesize well-aligned Si NW-CNT arrays exhibiting good electrochemical performances and high energy storage capacities for nanostructured anodes in Li-ion half cells^23^. One dimensional nanoscale heterojunctions of Si NW-CNT arrays, displaying enhanced electron field emission properties, have been also developed^24^.

In this paper we report a new and very simple route for embedding semiconducting SWCNTs in a Si NW array prepared by metal assisted wet etching. In the hybrid NW/CNT system both components maintain their optical activity, therefore the material acts as a very flexible light source exhibiting the unique feature of a simultaneous photon emission at room temperature both in the visible and in the IR regions. Although an analogous double emission has been previously observed in SiGe NWs^25^, in that case it was not possible to observe any Ge-related PL at room temperature. The great potentialities of the NW/CNT system for the realization of room temperature operating hybrid light sources are finally highlighted; indeed, the very wide range of emission spectra obtainable from this material makes it able to satisfy a wide range of different requirements for possible applications in Si photonics, including multiwavelength operation.

## Results and Discussion

Electronic and optical properties of SWCNTs are strictly related to their structure. Nanotube diameter and chiral angle are uniquely related to a pair of integers (*n*, *m*) that describe its construction as a rolled-up graphene sheet. Generally CNTs for which mod (*n–m*, 3) = 0 display metallic behavior; in the other cases, tubes have significant band gaps and show semiconducting behavior. The optical properties of semiconducting SWCNTs are ruled by excitonic recombination effects^26^. Optical emission occurs in the IR as a result of the absorption of one visible/UV photon bringing the system to the *eh*_22_ or *eh*_33_ excitonic levels, followed by fast non radiative decay to the fundamental *eh*_11_ level and radiative recombination of the electron-hole pair. Sharp PL peaks are only observed in isolated nanotubes or in small bundles, in which non-radiative decay channels occurring via exciton energy transfer towards metallic species are minimized^17^.

**Figure 1:**
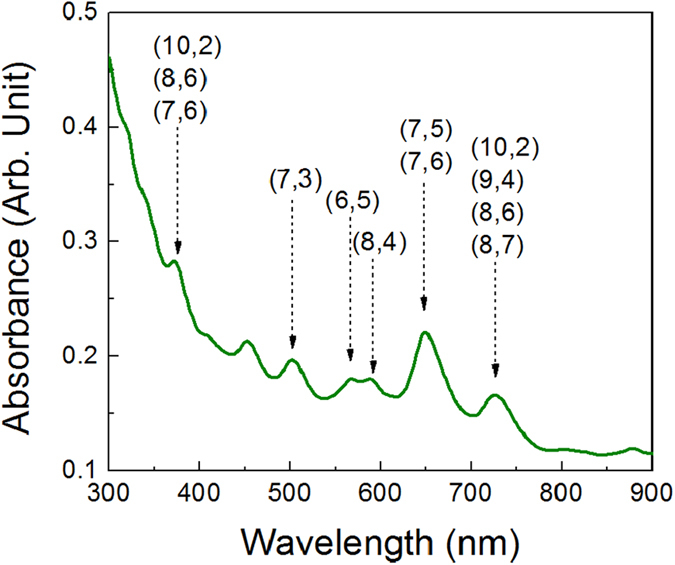
Absorption spectrum of a dispersion with a CNT concentration of about 0.5 mg/l. Peak assignment is based on the comparison with the data shown in Figs 2 and 3.

The optical properties of the CNT dispersion prepared according to the procedure described in the experimental section have been determined by using UV-VIS spectroscopy, PL and PL excitation (PLE) measurements performed at excitation wavelengths spanning within a wide range (300–750 nm). Data are presented in Figs 1 and refer to a dispersion with a CNT concentration of about 0.5 mg/l. Figure 1 shows the 300–900 nm range of a typical absorption spectrum; a series of peaks, due to the absorption of the many CNT chiralities present in the dispersion, is clearly visible, superimposed to a background due to the excitation of π plasmons, which becomes predominant at shorter wavelengths^27^.

**Figure 2:**
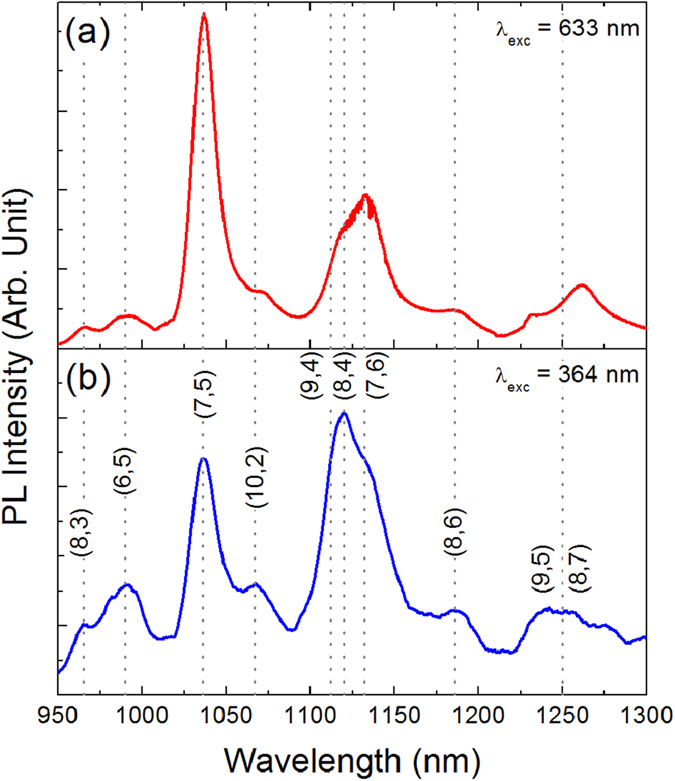
950–1300 nm range of the room temperature PL spectra of a dispersion with a CNT concentration of about 0.5 mg/l, obtained by exciting in the visible (at 633 nm, Figure 2a) and in the UV (at 364 nm, Figure 2b) regions. Peak assignment is based on the comparison with the data shown in Figs 1 and 3.

Figure 2 shows the 950–1300 nm range of the PL spectra of the dispersion, obtained by exciting in the visible (at 633 nm, Fig. 2a) and in the UV (at 364 nm, Fig. 2b) regions. Several sharp PL peaks, due to the emission of the different CNT families, can be recognized; basically, the same peaks are detected under both excitation modes, although relative intensities are quite different.

**Figure 3:**
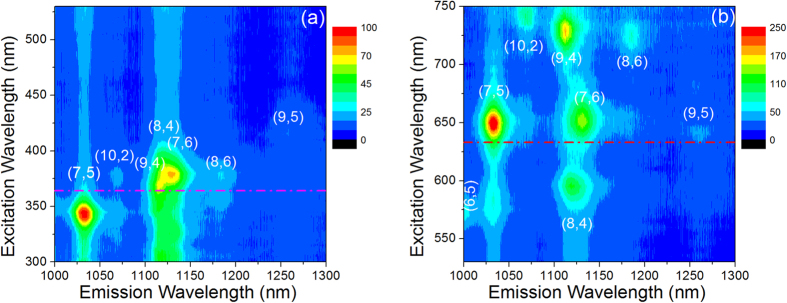
2D PLE maps plotting in a colour scale the PL intensity in the 1000–1300 nm range as a function of the excitation wavelength for a dispersion with a CNT concentration of about 0.5 mg/l; the excitation range 300–530 nm is shown in panel (**a**), while the range 530–750 nm is shown in panel (**b**).The dash-dotted red lines correspond to 364 and 633 nm excitation. Peak assignment is based on the comparison with the data shown in Figs 1 and 2.

Figure 3 shows the 2D PLE maps of the dispersion, plotting in a colour scale the PL intensity in the 1000–1300 nm range as a function of the excitation wavelength; the excitation range 300–530 nm is shown in Fig. 3a, while the range 530–750 nm is shown in Fig. 3b. The maxima in the maps correspond to the resonant excitation of the various CNT families.

The main CNT families present in the dispersion can be identified by cross-checking the absorption and emission data reported in Figs. 1. By looking at the short wavelength range of the PL spectra of Fig. 2, we can assign the emission peaks found at about 965, 990, 1035 and 1065 nm to the (8, 3), (6, 5), (7, 5) and (10, 2) CNT families, respectively. The broad emission peak located in the region between 1100 and 1150 nm contains contributions from three distinct families, i.e. (9, 4), (8, 4), and (7, 6), as clearly put into evidence by the presence of three well resolved excitation peaks in the map of Fig. 3b. Finally, at longer wavelengths we can identify the (8, 6) family, which emits at about 1185 nm, while the broad peak at around 1250 nm may include contributions from the (9, 5) and (8, 7) families. All assignments are in good agreement with the literature^18^,^28^,^29^.

From the analysis of the data in Figs 2 and 3, it is evident that a change of the excitation wavelength deeply influences the shape of the PL spectra of the CNT dispersion. By focusing on Fig. 2, we note that spectra obtained by exciting at 633 and 364 nm exhibit relevant differences in the relative intensities of the main emission peaks. This effect can be understood by considering the involved transitions. Indeed, in the case of 633 nm excitation only *eh*_11_ and *eh*_22_ transitions are involved so that the PL spectrum is dominated by peaks due to (7, 5) and (7, 6) families which resonantly absorb the exciting photons^18^,^28^, on the other hand, under 364 nm excitation, resonant absorption also inducing *eh*_33_ transitions occurs for the (10, 2), (7, 6) and (8, 6) CNT families^29^, which indeed exhibit an enhanced PL intensity. The spectrum of Fig. 2b describes a situation in which the emission from (7, 5) and that of the (9, 4), (7, 6) and (8, 4) families are roughly balanced, but the spectrum shape can be significantly changed even by small variation of the exciting wavelength. For instance, the analysis of Fig. 3a evidences that by reducing the excitation wavelength from 364 to about 340 nm, the emission of the (7, 5) family can be remarkably enhanced, becoming the dominant one, due to resonant absorption inducing *eh*_33_ transitions. Similarly, the contribution at about 1120  nm can become largely predominant over that of the (7, 5) family if the excitation wavelength is increased at about 380 nm, due to resonant absorption inducing *eh*_33_ transitions for the (7, 6) family. Analogous considerations can be done for visible excitation.

Note finally that both absorption and emission peaks are quite sharp, demonstrating that the procedure described in the experimental section for the preparation of the dispersion is very effective in avoiding the formation of large bundles. The major limitation for the use of CNT in photonics is, indeed, the formation of bundles due to van der Waals interactions between neighboring tubes. Bundles heavily affect the PL properties of CNTs, due to the occurrence of energy transfer among adjacent tubes. Since each bundle may contain both semiconducting and metallic CNTs, when energy transfer to metallic tubes occurs, light emission is quenched^30^.

**Figure 4:**
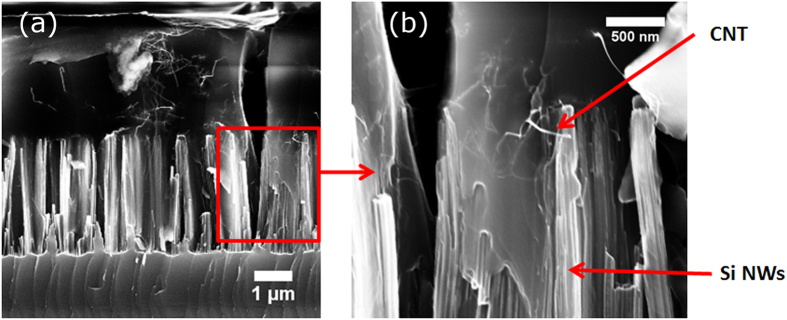
(**a**) SEM image of a typical NW/CNT sample. The NW mean diameter is 7 ± 2 nm, the length is about 3 μm and the density is about 10^11^ cm^−2^. CNTs are the long and thin structures which decorate the NWs. (**b**) SEM image showing at higher magnification a detail of the image reported in Figure 4a.

Hybrid NW/CNT samples have been synthetized according to the procedure described in the experimental section. Figure 4a reports a scanning electron microscopy (SEM) image of a typical Si NW array after infiltration with the dispersion with the highest CNT concentration. The mean diameter of the NWs is 7 ± 2 nm, as determined by structural characterization^6^, the length is about 3 μm and the density, as measured by plan view SEM images, is about 10^11^ NWs cm^−2^. In the image, CNTs are the long and thin structures which decorate the NWs. They are more clearly visible in the higher magnification SEM image reported in Fig. 4b. The comparison between the expected mean CNT diameter of about 0.9 nm and the size of the structures visible in the image (a few tens of nm) demonstrates that the CNT fraction visible by SEM actually mainly consists of small bundles. Smaller bundles or single tubes cannot be detected by SEM, so that their presence cannot be excluded a priori.

**Figure 5:**
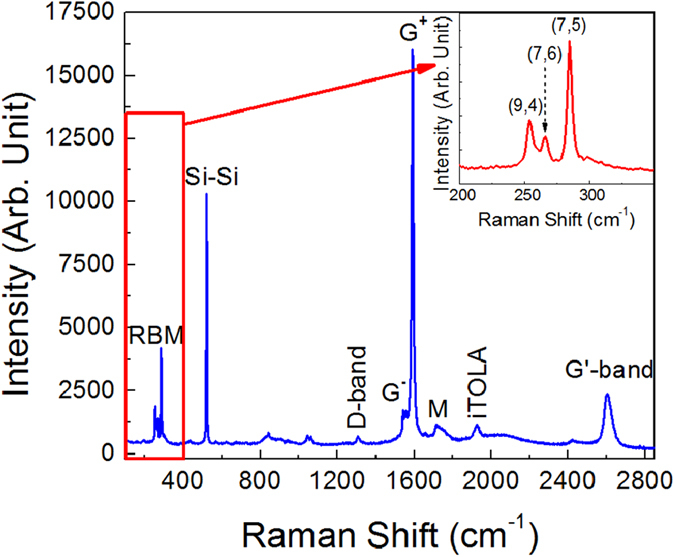
Raman spectrum, obtained by using an excitation wavelength of 633 nm, of a NW sample infiltrated with the dispersion with the highest CNT concentration. The main bands related to the SWCNT or Si NW vibration modes are identified. In the inset a detail of the spectrum, showing an expended view of the CNT RBM peaks, is reported.

The effective embedding of CNTs into the Si NW array is confirmed by the Raman spectrum shown in Fig. 5, obtained by using an excitation wavelength of 633 nm, referring to a NW sample infiltrated with the dispersion with the highest CNT concentration. Indeed, the spectrum shows the typical Raman bands related to the SWCNT vibration modes. The strongest bands that we observe are the radial breathing modes (RBM) and the G band, both first order Raman modes. In particular, in the low frequency range the three intense peaks centered at 253, 266 and 284 cm^−1^ can be assigned to the RBM of the (9, 4), (7, 6) and (7, 5) CNT chiralities, respectively^18^,^28^,^31^. These modes are referred to nanotubes only and are due to the bond-stretching phonon mode, out-of-plane, where all the C atoms move in phase along the radial direction; for instance, the frequency of a RBM is inversely proportional to the tube radius. The G band is split in more features having strong differences in intensities and shapes^31^. In particular, we notice that here it consists of two main contributions, peaked at about 1560 (G^−^) and 1590 cm^−1^ (G^+^), respectively; the first feature is due to in plane stretching vibrations of C atoms along the circumferential direction, the second is due to in plane vibrations along the axis of the tube^32^. Generally the G^−^ band becomes smaller for small tube diameters and it is strong and asymmetric for metallic tubes assuming a Breit-Wigner-Fano shape. In our case the Lorentian shapes for both G^−^ and G^+^ bands highlight the highly prevalent semiconducting nature of the detected CNTs. In Fig. 5 we observe also the presence of the D band at 1307 cm^−1^, a second order Raman scattering mode related to the disorder grade of the CNTs; its overtone, indicated as 2D or G’ band is peaked at 2604 cm^−1^ 33. Further vibrations coming from semiconducting tubes, the M band (an overtone mode at 1730  cm^−1^) and the iTOLA band (a combination of optical and acoustic modes at 1927 cm^−1^) are also shown in the spectrum^32^. Finally, the spectrum displays the first Raman peak of Si, centered at 521 cm^−1^ 34.

**Figure 6:**
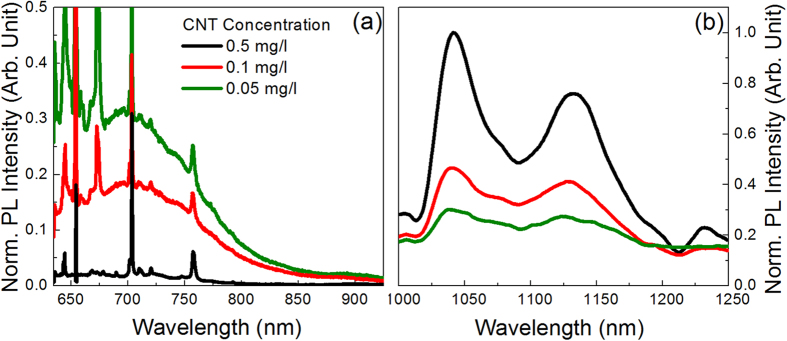
Room temperature normalized PL spectra of Si NWs infiltrated with dispersions characterized by three different CNT concentrations, obtained by exciting at 633 nm. (**a**) 635–900 nm region; (**b**) 1000–1250 nm region.

**Figure 7:**
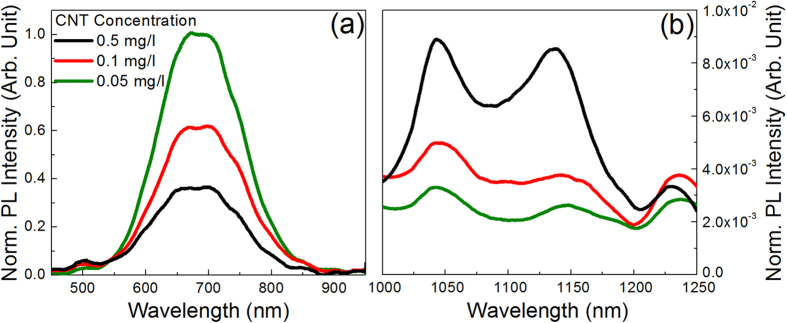
Room temperature normalized PL spectra of Si NWs infiltrated with dispersions characterized by three different CNT concentrations, obtained by exciting at 364 nm. (**a**) 450–950 nm region; (**b**) 1000–1250 nm region.

The room temperature PL spectra of Si NWs infiltrated with dispersions characterized by three different CNT concentrations have been obtained by exciting the system at 633 and 364 nm and are presented in Figs 6 and 7, respectively, after normalization to the maximum intensity. Noteworthy, intense signals both in the visible (see Figs 6a and 7a) and IR (see Figs 6b and 7b) regions are detected for both excitation wavelengths.

Figure 6a reports the emission spectrum in the 635–900 nm range obtained by exciting at 633 nm, which mainly consists of a wide band, centred at about 700 nm, due to electron-hole recombination in the NWs^6^,^8^; as expected on the basis of the very small NW diameter (about 7 nm), quantum confinement effects markedly blue-shift the emission with respect to that of bulk Si. The intense and sharp features superimposed to the NW emission are Raman peaks; the main ones are detected at about 645, 654, 672, 703 and 757 nm, and are assigned to the Si overtone scattering from acoustic phonons, Si first order Raman scattering, Si second order Raman scattering, G-band and G’-band of CNTs, respectively. The high intensity of the Raman peaks is due to the occurrence of multiple scattering phenomena; on the other hand, at the excitation wavelength of 633 nm Si NW absorption has a relatively low efficiency, even if the intensity of the PL signal remains strong enough to envisage practical applications. However, if a quantitative comparison between Raman and PL efficiencies is attempted, the very broad nature of the PL peaks has to be taken into account. Figure 6a demonstrates that by increasing the CNT concentration the intensity of the PL signal of NWs strongly decreases, becoming almost undetectable for the highest concentration, where essentially only Raman peaks are observed; some possible explanations for this effect will be discussed in detail hereinafter. No relevant variations of the PL peak position and shape are detected.

Figure 6b reports the 1000–1250 nm region of the room temperature PL spectra of the NW/CNT samples excited at 633 nm. Spectra are mainly characterized by the presence of two relatively broad peaks, centred at about 1042 and 1132 nm. On the basis of the comparison with the PL spectrum of the CNT dispersion reported in Fig. 2a, the feature at 1042 nm can be assigned to the convolution of the emission peaks due to CNTs with chirality (7, 5) and (10, 2), while that at 1132 nm to unresolved contributions from (9, 4), (8, 4) and (7, 6) chiralities. In addition, weaker peaks are found on the long wavelength side, mainly assigned to the (8, 6) and (9, 5) families. It is noteworthy that the hybrid system essentially exhibits the same PL peaks, with similar relative intensities, observed in the CNT dispersion for the same exciting radiation (see Fig. 2a). However, peaks of the hybrid samples appear remarkably broader than those of the CNT dispersion, indicating that the procedure for their synthesis implies the formation of larger bundles^30^. If we look at samples with lower CNT concentration we notice that, as expected, they exhibit a weaker PL signal, so that the low intensity peaks become almost undetectable; no relevant modification of the spectra shape are detected.

We have also studied the optical response of the NW/CNT system under UV excitation, where NW absorption is expected to be remarkably stronger than in the visible; indeed, for pure Si NWs we have measured under excitation at 364 nm a PL intensity which is a factor of 4 higher than that measured by exciting at 633 nm, for the same pump power. However, CNT absorption of 364 nm photons is not negligible; as above discussed for the case of the dispersion, resonant absorption to the *eh*_33_ levels occurs for some specific CNT families, which indeed, as shown in Fig. 2b, exhibit a remarkably enhanced PL intensity; furthermore, all CNT families exhibit an UV absorption due to the π plasmons.

The room temperature PL spectrum, measured by using an excitation wavelength of 364 nm, of hybrid samples characterized by different CNT concentrations is displayed in Fig. 7a,b. In particular, Fig. 7a reports the emission spectrum in the 450–950 nm region which, analogously to what shown in Fig. 6a for 633 nm excitation, consists of a wide band, centred at about 690 nm, due to electron-hole recombination in quantum confined Si NWs. Also in this case, the intensity of the PL signal of NWs decreases by increasing CNT concentration, although it remains quite strong even for the highest concentration; no relevant variations of the peak position and shape as a function of CNT concentration are detected.

Figure 7b reports the room temperature PL spectra of the hybrid samples in the 1000–1250 nm region. Spectra resemble those shown in Fig. 6b for 633 nm excitation, although the two most intense peaks (the one at about 1042 nm, due to (7, 5) and (10, 2) chiralities, and that at about 1132 nm, due to the (9, 4), (8, 4) and (7, 6) chiralities) have approximately the same intensity while the peak at shorter wavelength prevails under visible excitation. If we look at samples with lower CNT concentration we notice, as expected, a weaker PL signal, while no relevant modifications of the spectra shape are detected. Analogously to what shown in Fig. 3, a 2D PLE map for the hybrid system has been also obtained, and it closely resembles that of the dispersion.

**Figure 8:**
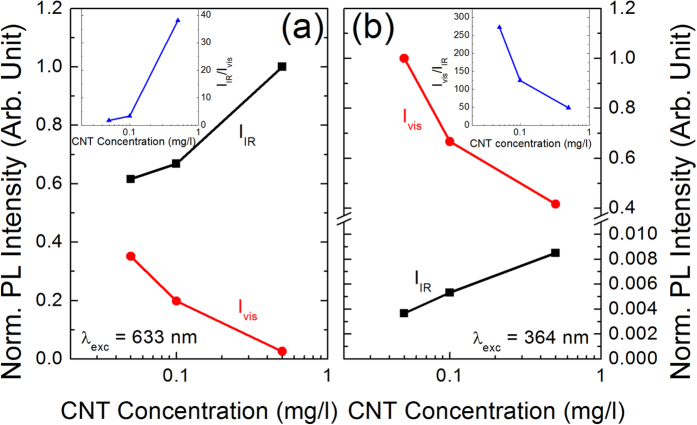
Normalized integrated PL intensities recorded in the visible (I_vis_) and in the IR (I_IR_) regions as a function of the CNT concentration for excitation wavelengths of (**a**) 633 nm and (**b**) 364 nm. The two insets report the ratio I_IR_/I_vis_ for Figure 8a and I_vis_/I_IR_ for Figure 8b as a function of the CNT concentration for the two excitation wavelengths.

Data in Figs 6 and 7 demonstrate that the NW/CNT system effectively behaves as a hybrid light source able to simultaneously emit photons in the visible (due to the NWs) and in the IR (due to the CNTs) regions at room temperature; furthermore, the ratio between the visible and the IR emissions can be varied within a wide range, by playing on the CNT concentration or on the excitation wavelength. Further insights about the potentialities of this system can be obtained from the quantitative analysis of the above reported PL spectra, although a comparison of the PL intensities is only possible between spectra obtained by using the same excitation source. Figure. 8 plots the normalized integrated PL intensities recorded in the visible (I_vis_) and in the IR region (I_IR_) as a function of the CNT concentration for the excitation wavelength of 633 nm (Fig. 8a) and 364 nm (Fig. 8b). By focusing on Fig. 8a, we notice that both PL intensities show a marked dependence on the CNT concentration: by increasing concentration, I_IR_ increases and I_vis_ decreases while IR emission prevails throughout the explored concentration range. The inset of Fig. 8a reports the ratio I_IR_/I_vis_ as a function of the CNT concentration, which demonstrates that I_IR_ can be up to a factor of about 40 stronger than I_vis_. While the increase of I_IR_ by increasing CNT concentration is a quite straightforward effect, the dependence of I_vis_ on the concentration is unexpected and deserves further discussions; from the analysis of Figs 1 and 3 we know that the 633 nm excitation is resonant with the (7, 5) and (7, 6) families, which therefore preferentially absorb the excitation beam; under these conditions an increase of CNT concentration may lead to a less efficient NW excitation, also because Si NWs, similarly to other Si nanostructures such as Si nanocrystals^35^, are very good light absorbers in the UV region, but their efficiency of light absorption is strongly reduced in the visible. Furthermore, we have also to take into account that photons emitted by NWs may be absorbed by the carbonaceous part of the hybrid system, causing a reduction of the PL external efficiency in the visible. Finally, the opposite behaviour of the visible and IR emission of the hybrid system by increasing CNT concentration could also account for the occurrence of a resonant energy transfer mechanism from excited NWs to neighbouring CNTs.

By focusing on Fig. 8b, we notice that also under 364 nm excitation both PL intensities show the same trends already found for the 633 nm excitation: by increasing CNT concentration, I_IR_ increases while I_vis_ decreases. Also in this case the decrease of I_vis_ by increasing CNT concentration, as previously discussed for the 633 nm excitation, may be due to CNT absorption of photons emitted by NWs or to an energy transfer mechanism; on the other hand, preferential absorption of incoming UV photons by CNTs is less probable, since NW are stronger absorber of UV photons than CNTs. In agreement with these considerations, the maximum CNT-induced decrease of I_vis_ accounts only for about a factor of 2 at 364 nm, while it account for about a factor of 15 at 633 nm. The inset of Fig. 8b reports the ratio I_vis_/I_IR_ as a function of the CNT concentration; NW-related visible emission remains largely prevalent even for the highest concentration and it can be up to a factor of about 300 stronger than that of CNT for the lowest concentration. Since, as previously discussed, the enhancement of the PL intensity of pure NWs on moving from 633 to 364 nm excitation accounts for about a factor of 4, the much more larger enhancement of the visible emission with respect to the IR one exhibited by the hybrid system must be also due to a strong reduction of the CNT absorption.

## Conclusions

We have demonstrated a very simple route for the effective and stable embedding of semiconducting SWCNTs in a Si NW array. In the resulting system both components remain optically active, therefore NW/CNT samples act as a very flexible hybrid light source exhibiting the unique feature of a simultaneous photon emission at room temperature both in the visible (owing to NWs) and in the IR (owing to CNTs) regions.

A crucial advantage of using the NW/CNT hybrid instead of the two single materials is a greater compatibility with Si technology. Indeed, while pure CNT films can be hardly handled in an industrial environment, the hybrid system essentially behaves as a pure NW array, leading to an easier processing. Moreover the hybrid may allow the realization of a more compact and size-scalable light source, owing to the simultaneous excitation of both materials with the same source.

Another remarkable feature of the hybrid material is the possibility to vary the I_vis_/I_IR_ ratio within 4 orders of magnitude by changing the CNT concentration or the excitation wavelength. Furthermore, while the shape of the NW-related emission remains almost unchanged by varying the excitation wavelength, the shape of the IR emission changes when the exciting radiation is able to resonantly excite some of the CNT families contained in the mixture infiltrated in the NW array. This means that, simultaneously to the variation of the I_vis_/I_IR_ ratio, by properly selecting the excitation wavelength, the IR emission can be modulated in order to enhance the intensity of selected PL peaks. We finally underline that the visible or the IR emissions can be also modulated by changing the NW size or the composition of the CNT mixture, respectively.

The data we have presented demonstrate the great potentialities of the NW/CNT system for the realization of multiwavelength, hybrid light sources operating at room temperature; the very wide range of emission spectra obtainable from this material makes it able to satisfy a wide range of specific requirements for possible applications in Si-compatible photonics.

## Methods

Si NWs were obtained by starting from n-type, single crystal, (100)-oriented Si wafers. Wafers were UV oxidized and dipped in 5% HF to obtain clean and oxide-free Si surfaces. Then an ultrathin Au layer, 2 nm thick, was deposited at room temperature by electron beam evaporation on the Si samples, by using high purity (99.99%) Au pellets as a source. Finally, Au coated samples were etched at room temperature in an aqueous solution of HF (5 M) and H_2_O_2_ (0.44 M) to form Si NWs^6^. SWCNTs were purchased from SouthWest NanoTechnologies (SWeNT SG76, Lot #SG76-L29), under the form of a mixture of several chiralities, most of which exhibits a semiconductor behavior. As purchased material consists of bundles of CNTs having a mean length of 700 nm and a median diameter of about 0.93 nm. For the debundling of the SWCNTs we followed a standard procedure^36^; 2 mg of CNTs were added to 10 ml of deionized H_2_O containing 0.2 g of taurodeoxycholic acid sodium salt hydrate, acting as a surfactant. The solution underwent ultrasonic treatment for 30 min in a horn sonifier (Branson 250) and was then centrifuged with an Optima Max-XP tabletop ultracentrifuge, equipped with a swinging bucket rotor MLS-50, operating at 45000 rpm for 45 min. The surnatant, consisting of well-dispersed individual CNTs and small bundles, is finally extracted, while larger bundles and insoluble impurities are removed^36^. According to what reported in literature for a similar preparation procedure^17^, we estimate that the obtained dispersion has a CNT concentration of about 0.5 mg/l. In order to synthesize the hybrid NW/CNT system, 5 μl of the dispersion were drop-casted on a 0.5 × 1 cm^2^ NW sample; following air exposure at room temperature, CNTs are stably embedded in the NW array. NW/CNT samples containing a lower amount of tubes were prepared by using less concentrated dispersions, obtained by diluting 5:1 (v/v) (corresponding to a concentration of 0.1 mg/l) or 10:1 (v/v) (concentration of 0.05 mg/l) the starting one.

The optical characterization (PL and Raman scattering) of the CNT dispersions and of CNT-infiltrated Si NW samples was performed at room temperature by using a Horiba Jobin Yvon HR800 Micro-Raman spectrometer, coupled with an Ar^+^ laser line, tuned at 364 nm and operating at pump power up to 0.38 mW, and a He-Ne laser line, operating at pump power up to 7.8 mW. Both excitation sources were focused on the samples through a 50 × LWD (NA = 0.5) or a 100 × (NA = 0.9) objective. Raman and PL spectra were acquired in the visible range by using a Peltier-cooled CDD detector (Synapse), while an InGaAs array N_2_-cooled detector (Symphony II) was used for the IR range. Absorbance measurements were performed by using a PerkinElmer Lambda 20 spectrometer, while PLE maps were acquired by using a NanoLog spectrofluorimeter, equipped with a Xe lamp and an InGaAs N_2_-cooled detector. SEM images were obtained by using a Gemini Field Emission SEM Carl Zeiss SUPRA 25.

## Additional Information

**How to cite this article**: Lo Faro, M. J. *et al.* Silicon nanowire and carbon nanotube hybrid for room temperature multiwavelength light source. *Sci. Rep.* **5**, 16753; doi: 10.1038/srep16753 (2015).
